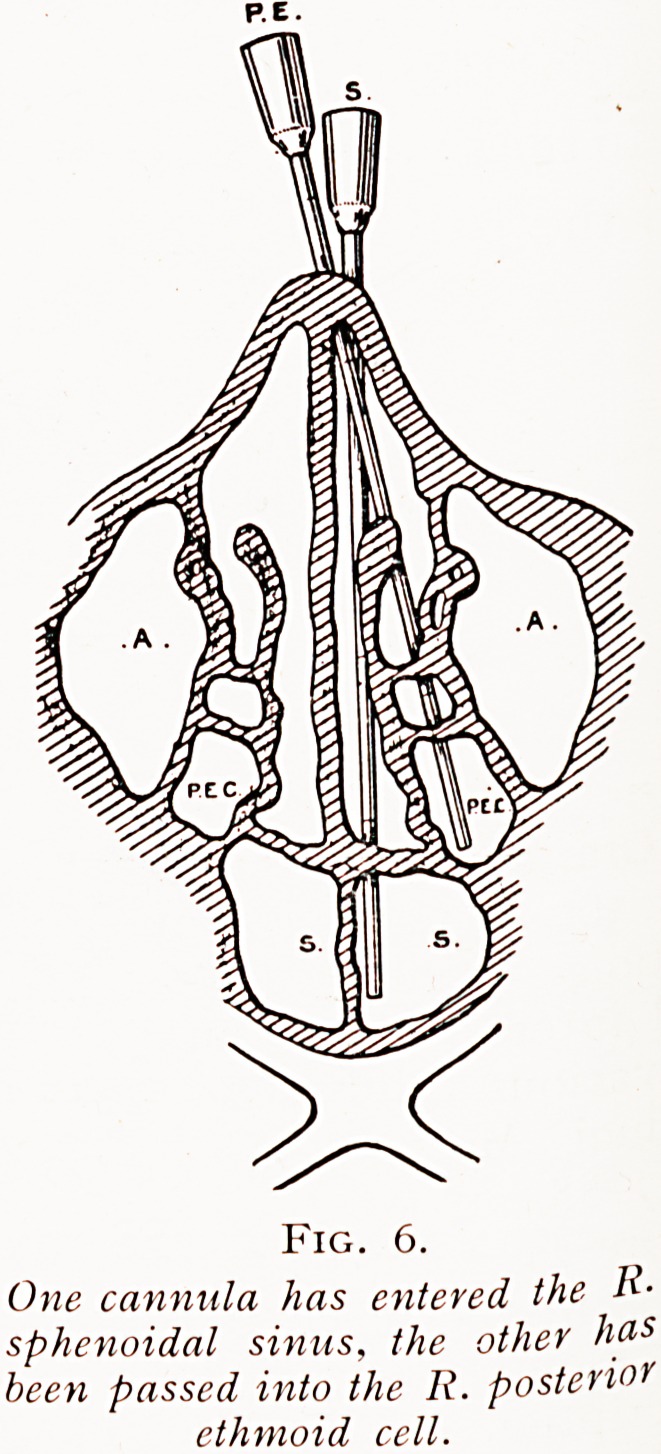# Some Points in the Differential Diagnosis of Focal Infection in the Nasal Sinuses Causing Ocular and Other Systemic Infections
1A Paper read at a Meeting of the Bristol Medico-Chirurgical Society, on Wednesday, April 8th, 1925.


**Published:** 1925

**Authors:** Patrick Watson-Williams

**Affiliations:** Consulting Surgeon for Diseases of Ear, Nose and Throat, Bristol Royal Infirmary


### SOME POINTS IN THE DIFFERENTIAL DIAGNOSIS OF FOCAL INFECTION IN THE NASAL SINUSES CAUSING OCULAR AND OTHER SYSTEMIC INFECTIONS.[1]

BY

PATRICK WATSON-WILLIAMS, M.D. Lond.,

*Consulting Surgeon for Diseases of Ear, Nose and Throat, Bristol Royal Infirmary.*

THE purport of these notes is not to survey the question of nasal infections as the causal factor in various systemic affections, nor even to discuss the manifold symptoms attributable to such focal infection, but to explain briefly some developments in technique for locating precisely, or positively excluding, the existence of a nasal source of infection in cases where the consequences of focal infection render it a matter of considerable importance to obtain reliable diagnostic findings, *e.g.* canalicular optic neuritis and other peripheral neuritis, rheumatoid arthritis, neurasthenia, mental depression and even insanity.

The existence of frontal sinusitis in doubtful cases may usually be diagnosed by means of good skiagrams and further corroborated by passing a fine cannula into the sinus and blowing air so as to cause any secretion from the sinus to appear at the lower end of the fronto-nasal duct.

In antral sinus infection transillumination is so unreliable as to be useless and often misleading and, though skiagraphy is often valuable, the less marked examples can only be determined with precision by entering the sinus with a

[1] A Paper read at a Meeting of the Bristol Medico-Chirurgical Society, on Wednesday, April 8th, 1925.

cannula and sucking the contained secretion into a sterile syringe for examination.

It is, however, in determining and exactly localising the focus of infection when present in the sphenoidal sinuses or posterior ethmoidal cells that the suction syringe has proved most helpful. Investigations in the post-mortem room which I carried out in 1896 with the co-operation of Mr. S. V. Stock led me to introduce a method of puncturing the anterior wall of the sphenoidal sinus and sucking any contained discharge back into the attached sterile syringe, instead of syringing out the sinus by the natural opening. Subsequently I applied the same method to the posterior ethmoidal cells, and thus with the suction syringe one can determine the presence or absence of active infective processes in either the maxillary antrum, sphenoidal sinuses and anterior or posterior ethmoidal cells on either side.

Let us take for example a patient threatened with loss of vision from canalicular neuritis, suspected as nasal in origin. Having explored various sinuses and found them sterile, it is necessary to make certain that no infected sinus has been "missed," because the failure to locate exactly the infected focus may cost the patient his eyesight, if nothing worse. Further, if some of the sinuses have been proved infected, it does not follow that they are the chief or sole source or even the source at all of a canalicular neuritis, unless one can be certain that the other sinuses which might cause the neuritis have been explored and proved sterile.

Diagnostic exploration by the suction syringe can be done under local anæsthesia by cocaine, tutocain or eucaine, etc., in all but children or highly nervous adults for whom a light general anæsthetic is desirable.

For each sinus explored one should use a separate sterile syringe and cannula. After entering a sinus a little sterile warm water may be injected to mix with any discharge lying in the cavity, and then sucked back into the syringe, yielding, so to speak, a "deep-sea fishing" sample of the sinus contents. After detaching the cannula, the contents of the syringe is emptied into a sterile bottle for examination by stained film and culture.

The maxillary antrum may be entered either through the thin bone of the upper part of the inferior meatal wall

FIG. 1.

*Diagram with left nasal passage exposed to show exploration of left antrum through the middle meatus.* (1) *First position, the curved end of cannula placed in the middle meatus.* (2) *Second position prior to passing cannula down and outwards into the antrum on raising the hand and syringe upwards.*

FIG. 2.

*Showing above the cannula passed into the maxillary antrum through the middle meatus, and below the same with the short cannula through the thin inferior antro-meatal wall.*

or through the far thinner wall of the middle meatus (as shown in the diagram), and to anyone accustomed to the latter route it is a very simple and painless method.

*The sphenoidal sinus* is entered by passing the blunt trocar and cannula through the thin anterior wall, care being taken to avoid using any force. The sensation of resistance overcome indicates the entry of the top of the cannula into the sinus, and it is passed until it impinges against the posterior wall of the sinus ; the sensation of contact with a bony wall being characteristic and hardly mistakable. Having thus entered the sphenoidal sinus cavity, the blunt trocar is withdrawn and the syringe attached to the proximal or projecting end and the sample of the investigated sinus content sucked into the syringe. If much pus is lying in the

FIG. 3.

*Transverse horizontal section of the head showing the floor of the intracranial anterior fossa. Posteriorly the optic chiasma divides, and the right optic nerve is displayed passing forwards to the right eye and skirting the small right sphenoidal sinus and posterior ethmoidal cell. Exploring cannulæ here shown entering the right and left nasal passage, but their distal extremities are seen lying side by side in the large exposed left sphenoidal sinus.*

sinus, it may appear in the syringe in ropes, or as very bloody mucus, leaving one in no doubt as to the sinus being infected. In other cases the fluid extracted may be perfectly clear and watery, proving sterile on culture. Other samples obtained may be suspicious only, and doubtful until submitted to film and culture examinations, which may either prove negative or definite phagocytosis, abundance of polymorphonuclears and pyogenic cocci may afford conclusive evidence of active infection.

Fig. 4.
*Showing the exploratory cannula passed into the right posterior ethmoidal cell through the superior meatus.*—(*P.W.W.*)

*The posterior ethmoidal cells* are similarly explored by the blunt trocar and cannula entered either above and internal to the middle turbinated body, or, as I usually find much simpler, by piercing the posterior ethmoidal cell walls beneath the attachment of the middle turbinal body to the outer wall of the nasal passage.

Having thus explored the maxillary antrum, ethmoidal cells and sphenoidal sinuses through the right and left nasal passages, one has obtained at least six samples for investigation by the pathologist, which if the anatomical arrangement of the sinuses be normal should yield exact and reliable information as to the normal or infected condition of each sinus.

Unfortunately, the sphenoidal sinuses and ethmoidal cells are often most irregularly developed. For instance, one sphenoidal sinus may be very large and extend right across the mid-line, pushing the sinus of the other side, which is correspondingly small, so far outwards that the exploring cannulæ passed backwards through the right and left nasal passages have to be entered through the one large sinus, the other small one being missed (*vide* Fig. 3). This is not difficult to discover if, with both cannulæ in the sphenoidal sinus or

FIG. 5.
*Diagram showing the cannulæ passed through the R. and L. nasal passages and lying in the corresponding sphenoidal sinuses.*

FIG. 6.
*One cannula has entered the R. sphenoidal sinus, the other has been passed into the R. posterior ethmoid cell.*

sinuses, one injects fluid down one, for if both cannulæ be in one and the same cavity the fluid will rise up the other and escape by the open end, whereas if the two cannulæ are in separate sinuses the fluid injected down one cannula cannot escape by the other. Similarly a posterior ethmoidal cell may be so large and extend backwards so deeply that it encroaches on the corresponding sphenoidal sinus, and then one may be left in doubt as to whether one has entered the sphenoidal sinus instead of the ethmoidal cell. By again keeping both cannulæ *in situ* and injecting water down one it is quite easy to determine whether the two cannulæ lie in the same or in separate cavities. Such precautions are by no means fanciful, and I have met with cases of canalicular optic neuritis where the sinus infection did not correspond with the retinal defects until further careful exploration revealed such irregularities as I have described, and which had very nearly led to the source of the very serious ocular defect being missed and the patient unrelieved.

## Figures and Tables

**Fig. 1. f1:**
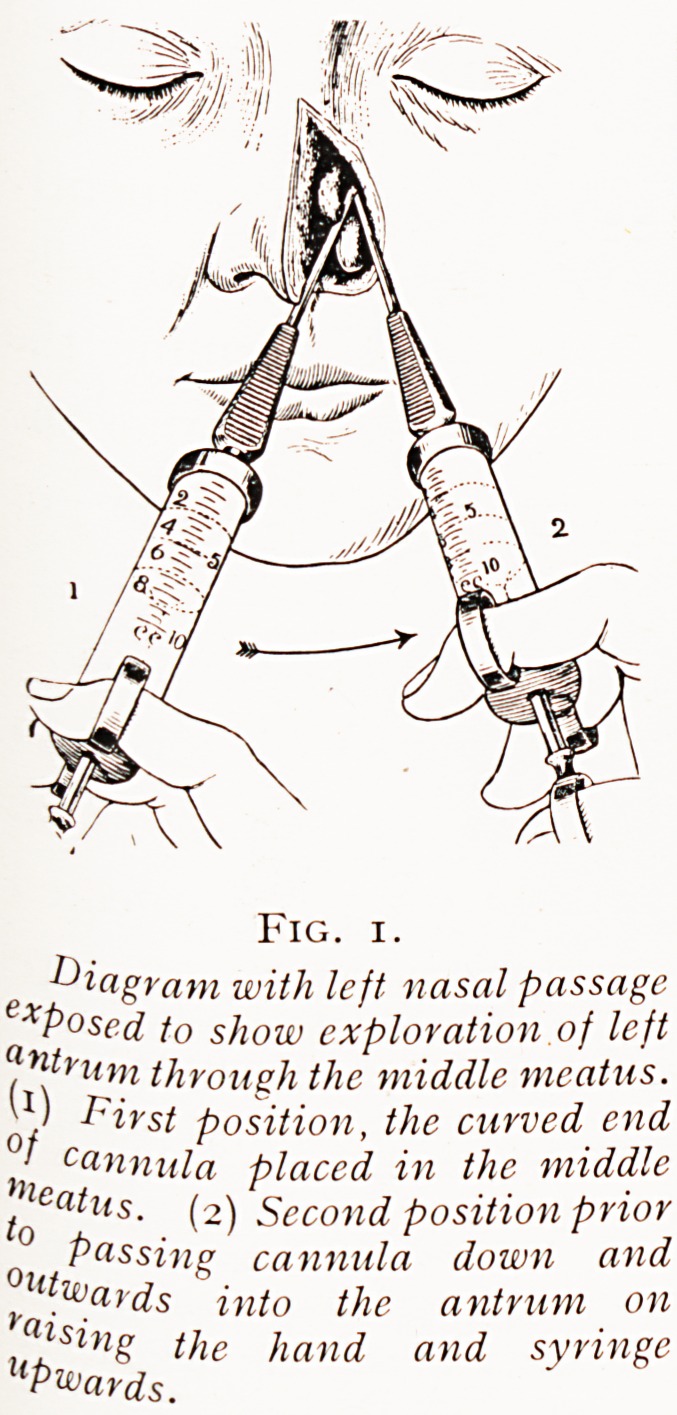


**Fig. 2. f2:**
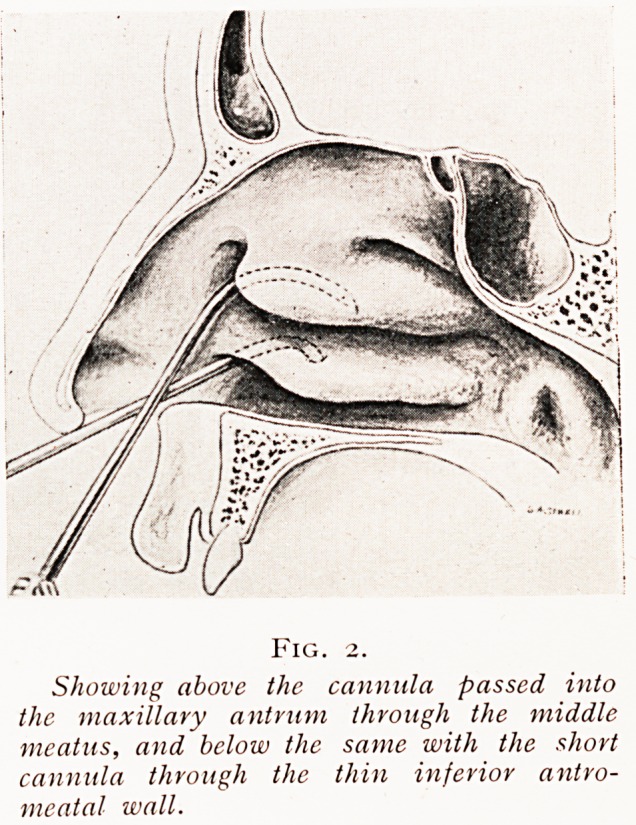


**Fig. 3. f3:**
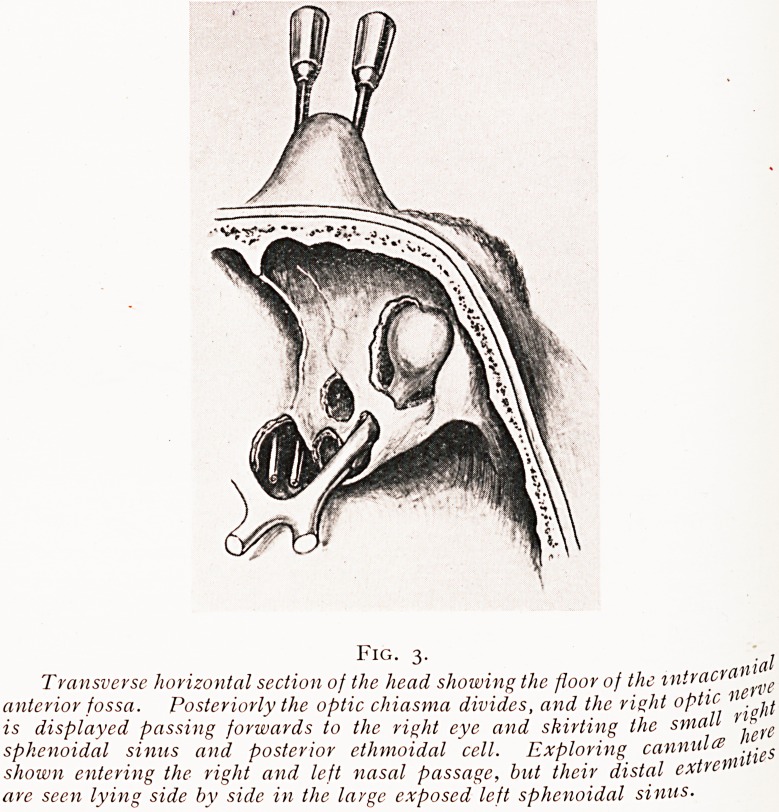


**Fig. 4. f4:**
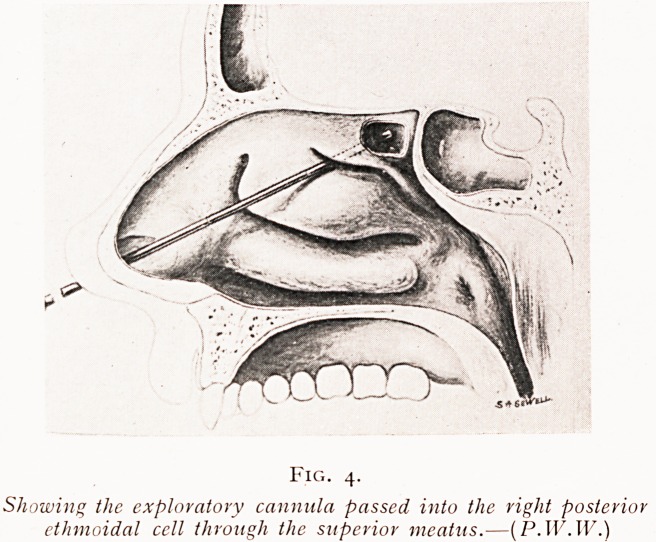


**Fig. 5. f5:**
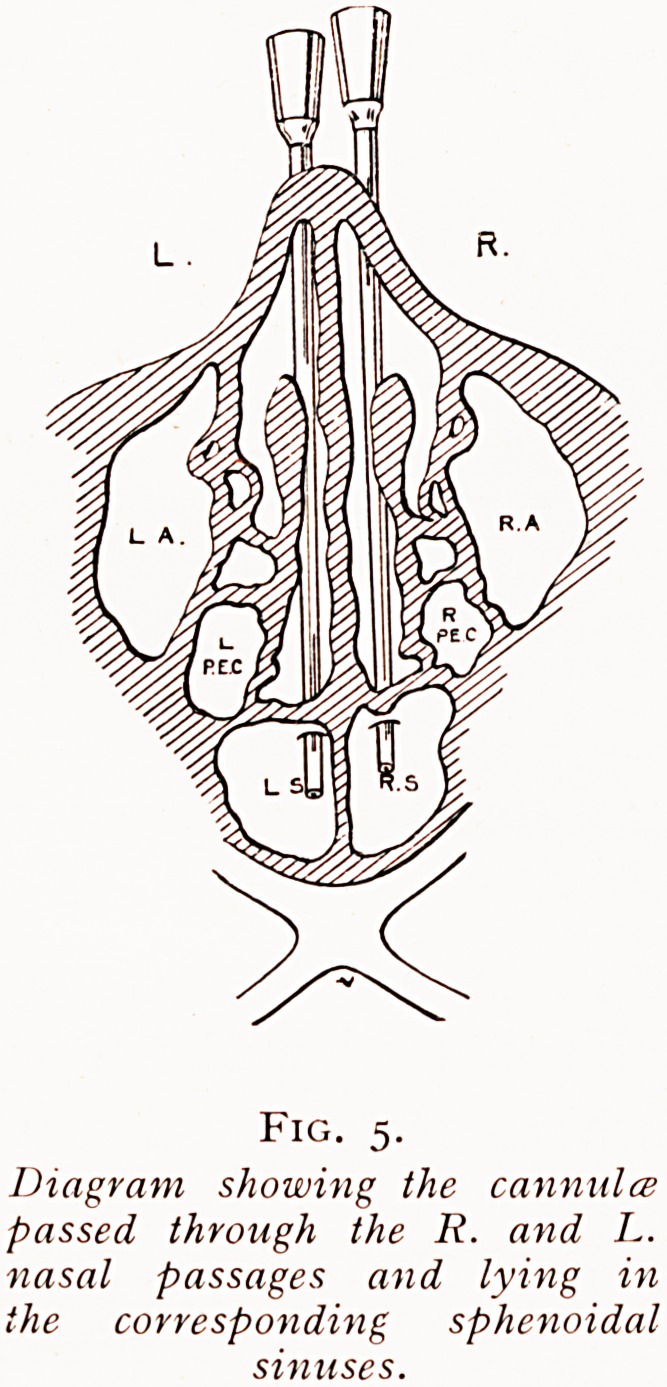


**Fig. 6. f6:**